# Abnormal Functional Connectivity of the Primary Sensory Network in Autism Spectrum Disorder: Sex Differences, Early Overdevelopment, and Clinical Significance

**DOI:** 10.1002/brb3.70363

**Published:** 2025-03-23

**Authors:** Yanan Zhang, Quan Zhou, Limei Gao, Jingwen Li, Hong Li, Gong‐Jun Ji, Hua Yang, Enze Wang, Kai Wang, Dandan Li

**Affiliations:** ^1^ School of Mental Health and Psychological Sciences Anhui Medical University Hefei China; ^2^ First Clinical Medical College Anhui Medical University Hefei China; ^3^ Anhui Hospital Affiliated to the Pediatric Hospital of Fudan University (Anhui Provincial Children's Hospital) Hefei China; ^4^ Collaborative Innovation Center of Neuropsychiatric Disorders and Mental Health Hefei China; ^5^ Department of Neurology The First Affiliated Hospital of Anhui Medical University Hefei China; ^6^ Research Center for Translational Medicine The Second Hospital of Anhui Medical University Hefei China

**Keywords:** autism spectrum disorder (ASD), early overdevelopment, functional connectivity (FC), primary sensory cortex

## Abstract

**Introduction:**

Primary sensory processing is atypical in patients with autism spectrum disorder (ASD) and affects daily functioning. However, the functional connectivity (FC) patterns of primary networks in ASD have not been systematically investigated.

**Methods:**

Primary networks were defined as four regions of interest (ROIs) in each brain hemisphere. We analyzed ROI‐wise FC in 105 individuals with ASD and 132 typically developing (TD) participants from Autism Brain Imaging Data Exchange I. We calculated the correlation between abnormal FC and clinical scores. Additionally, data from 53 individuals with ASD from our laboratory's two‐site dataset were used to validate the results and assess the effects of sex and age on FC consistency.

**Results:**

Regarding the ROI‐wise connectivity, significant group differences in FC emerged in several regional pairs, particularly in the primary auditory and somatosensory regions. Abnormal brain regions correlated with clinical symptoms. As age increased, abnormal FC had an initial fast and then slowing development trend, and the abnormal FC in females was higher than that in males. The two‐site dataset results were consistent with those of the multisite dataset in assessing the influence of age and sex on FC.

**Conclusion:**

Abnormal FC exists in the primary sensory cortex of patients with ASD, which correlates with clinical outcomes and may cause impairments in advanced cognitive functions. In addition, the primary sensory cortex of patients with ASD may undergo excessive growth in the early stages and demonstrate imbalanced development according to sex. These findings may help identify new biomarkers for ASD.

## Graphical abstract

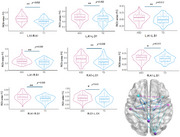

## 1 Introduction

Autism spectrum disorder (ASD) is a complex neurodevelopmental disorder characterized by deficits in social communication and the presence of restricted, repetitive behaviors (Ren et al. 2023). Most ASD research has focused on the higher cognitive functions of individuals with ASD (e.g., joint attention, social cognition, or repetitive behaviors) (LeBlanc and Fagiolini 2011). However, the development and proper execution of higher cognitive processes may depend on normal primary processing (Martínez et al. 2020). Primary processing requires information obtained concurrently from several sensory areas (Hong et al. 2019). For example, communication and socialization involve parallel auditory (Wilson et al. 2022), visual (Stickel et al. 2019), and somatosensory information processing. Nonetheless, atypical hierarchical information processing may hinder sensory and social functioning in individuals with ASD (Cechmanek et al. 2018; Knill and Pouget 2004). Research suggests that underutilization of top‐down processes, such as context or experience, or overreliance on bottom‐up sensory perception occurs in ASD (Thye et al. 2018).

Sensory processing abnormalities in ASD are reported in patients of all ages and levels of symptom severity (Park et al. 2021) and adversely affect both daily functioning and academic performance (Baum et al. 2015; Howe and Stagg 2016). Such abnormalities have been documented across all sensory modalities, and up to 95% of parents of children with ASD report some atypical sensory behaviors in their children (e.g., seeming indifference to pain, avoidance of certain sounds or textures, and sensitivity to certain odors) (Baird et al. 2003; Rogers and Ozonoff 2005; Thye et al. 2018). Dysfunctional connectivity may lead to atypical sensory behaviors in ASD (Park et al. 2021). How intrinsic connectivity is altered in the autistic brain is debated within the resting‐state functional magnetic resonance imaging (rs‐fMRI) literature, with reports of both over‐ and underconnectivity (Hull et al. 2016). Ilioska et al. (2023) reported that underconnectivity in autism is most prevalent for functional connections within the somatomotor network and connections linking the visual and somatomotor networks to each other and to attentional networks. However, Cerliani et al. (2015) indicated that there is overconnectivity between primary sensory regions and subcortical regions, with overall connection strength positively correlated with ASD symptom severity. They reported that overconnectivity between the sensory and subcortical regions is a central feature of ASD. These discrepancies in research findings indicate that the connectivity patterns of the primary sensory network require further exploration.

Local connectivity intensification in the primary sensory network of individuals with ASD has been proposed to generate delays in the perceptual pipeline and information transmission to subsequent systems, compromising the integration of information (Wass 2011). Successful integration of sensory inputs is crucial for both basic perceptual functions and higher order processes related to social behavior and cognition (Martínez et al. 2020). Deficits in sensory integration may impair the early stages of information processing with cascading effects on higher order social and cognitive functions, which are collectively associated with deficits in language development and social interaction (Baranek et al. 2013). Increasing evidence suggests that abnormal connectivity patterns are a hallmark feature of ASD neuropathology (Rausch et al. 2016). As a widely employed analytical method for rs‐fMRI, functional connectivity (FC) is useful for detecting abnormal interactions of brain regions (Wang et al. 2020). Regions of interest (ROI)‐wise quantitative features extracted from fMRI can mirror brain region connectivity and have the potential to serve as biomarkers of ASD and assist in clinical assessments (Wang et al. 2023). Thus, this study systematically explored the FC patterns of the primary sensory network in patients with ASD and how such abnormal patterns act on higher order cognitive networks.

Recent studies demonstrated that ASD is an age‐ and sex‐related neurodevelopmental disorder. The early brain overgrowth theory (EBO) hypothesizes that children with ASD experience excessive brain development in early life, which may lead to abnormal FC patterns (Cerliani et al. 2015). Nomi and Uddin (2015) discovered that children demonstrate overconnectivity patterns, adolescents show little underconnectivity, and adults with ASD display normal connectivity. Hormones invariably play a key role in sex differences (Williams et al. 2021). Ovarian hormones may also regulate brain development and neural connections. For example, they may influence the normal development of brain regions related to social cognition and emotional processing, manifesting as atypical connectivity patterns (Beltz and Moser 2020). Zhou et al. (2024) discovered that females with ASD have stronger right frontoparietal network–default mode network (DMN) connectivity than males with ASD. Notably, current research focuses more on social functional networks related to advanced cognition (such as the default network, salience network, and reward circuit), neglecting the systematic study of primary sensory networks (Hong et al. 2019).

The present study aimed to systematically explore the FC patterns of the primary sensory network in ASD using a large‐scale multisite dataset (Autism Brain Imaging Data Exchange, ABIDE) and to evaluate the effects of sex and age on abnormal FC. We verified the influence of sex and age using a two‐site dataset collected in the laboratory. In the current study, we extracted eight seed points from the primary visual cortex V1 (Brodmann BA17), primary auditory cortex A1 (BA41), primary somatosensory cortex S1 (BA1, BA2, and BA3), and primary olfactory cortex O1 (BA13, 14, 15, and 16) and calculated the resting‐state FC related to each ROI in the primary sensory network. Subsequently, we explored the relationship between abnormal FC and clinical symptoms and investigated the effects of sex and age on abnormal FC. Finally, for further validation, we used data from 53 individuals with ASD from two small datasets collected by our laboratory at two centers to conduct ROI‐wise FC analysis and assessed whether the effects of sex and age on FC were consistent. We hypothesized that (1) individuals with ASD exhibit altered FC in the primary sensory network compared with typically developing (TD) individuals. (2) Atypical connectivity is associated with clinical symptoms in ASD. (3) Age and sex affect atypical connectivity. (4) The results of the two‐site dataset are consistent with the influence of sex and age on FC evaluated in the multisite dataset.

## 2 Materials and Methods

### 2.1 Participants

In this study, we used the publicly available ABIDE I dataset (http://fcon_1000.projects.nitrc.org/indi/abide/) (Di Martino et al. 2014). It provides structural and rs‐fMRI images as well as demographic information of 1112 subjects from 17 sites (539 individuals with ASD and 573 TD individuals; age range: 7–64 years; median age: 14.7 years).

Subjects were excluded according to the following criteria: (1) absence of functional or structural images; (2) lack of a full‐scale Intelligence Quotient (FIQ) score or an FIQ score <70; (3) severe artifacts or signal losses in the fMRI data as visually inspected by two authors; (4) head motion >3 mm or >3°; (5) poor spatial normalization as visually inspected by three authors; and (6) scanning coverage <90% of the whole‐brain mask. Finally, 237 participants, including 105 individuals with ASD and 132 TD individuals (age range: 7–25 years) from 8 sites, were included in the data analysis.

### 2.2 fMRI Data Processing

Functional images were preprocessed using the WhiteMatterSF toolkit (Ji et al. 2017) (https://github.com/jigongjun/Neuroimagingand‐Neuromodulation) (Ji et al. 2017), which encompasses SPM12 (http://www.fil.ion.ucl.ac.uk/spm/software/spm12), AFNI (Cox 1996), and FSL (http://fsl.fmrib.ox.ac.uk/fsl) (more detail about MRI data acquisition in Table S1). The preprocessing steps included (1) removal of the initial five volumes; (2) slice timing correction and realignment; (3) co‐registration of individual functional data with structural images, segmenting structural images into gray matter, white matter (WM), and cerebrospinal fluid (CSF); (4) regression analysis to remove interfering signals, incorporating 24 head motion parameters and mean signals from the whole brain, WM, and CSF; (5) smoothing of functional images using a 4‐mm isotropic Gaussian kernel; and (6) temporal bandpass filtering (0.01–0.1 Hz). Images with head movements >3 mm of translation or >3° of rotation during fMRI data acquisition were excluded.

### 2.3 Seed Selection and Functional Connectivity Analysis

**FIGURE 1:**
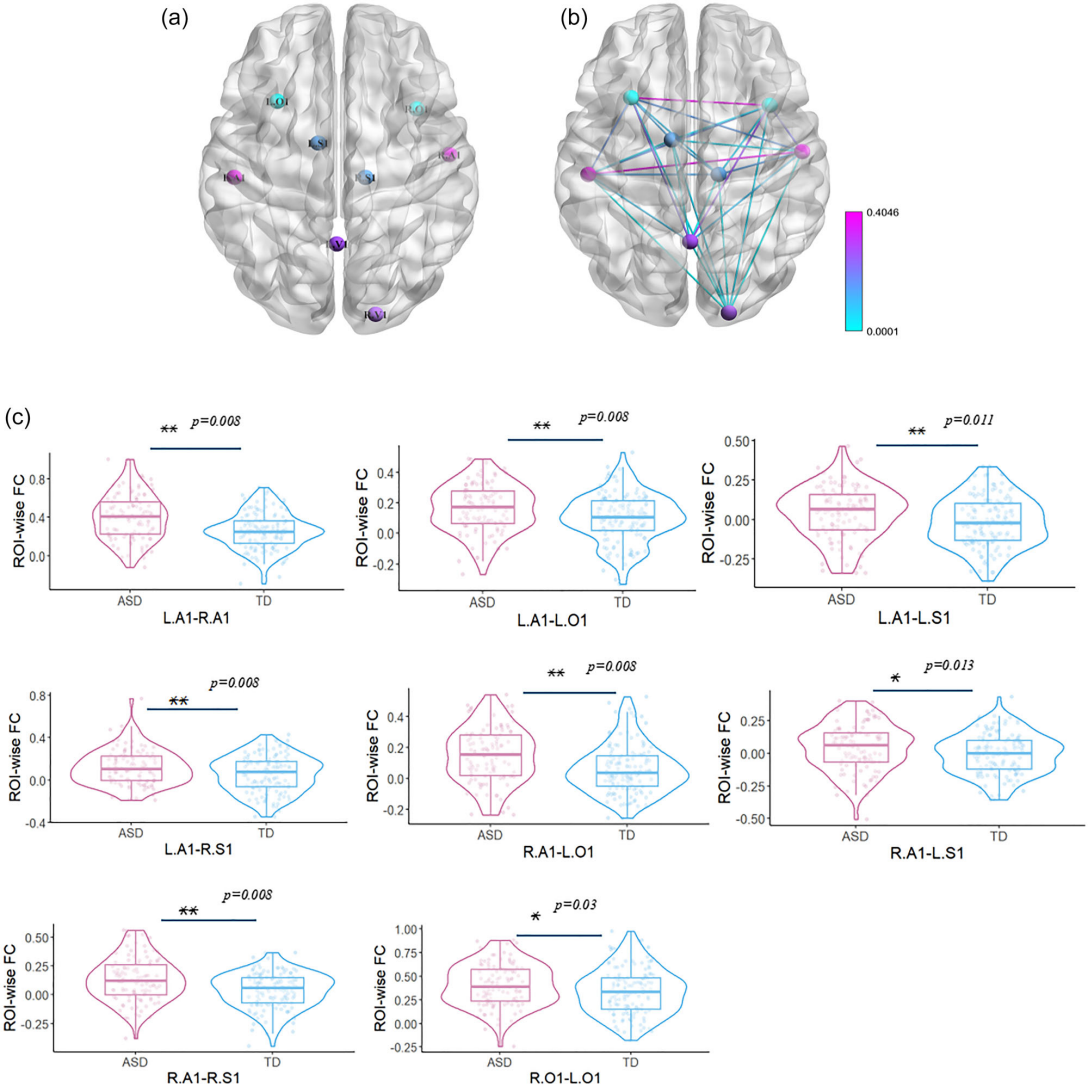
**Aberrant ROI‐wise FC**: (a) ROI definition: L.A1, left primary auditory cortex; R.A1, right primary auditory cortex; L.V1, left primary visual cortex; R.V1, left primary visual cortex; L.S1, left primary somatosensory cortex; R.S1, right primary somatosensory cortex; L.O1, left primary olfactory cortex; R.O1, right primary olfactory cortex; (b) FC between ROIs with significant group differences. (c) Significant ROI‐wise FCs. Boxes represent the median and interquartile range with whiskers indicating minimum and maximum values (pink for ASD; blue for TD). The violin plot outlines illustrate kernel probability density (FDR correction. *p < 0.05; **p < 0.01). ASD, autism spectrum disorder; FC, functional connectivity; FDR, false discovery rate; ROI, regions of interest; TD, typically developing.

On the basis of previous studies, eight classical primary sensory network ROIs (bilateral V1, A1, S1, and O1) were selected. ROIs were defined as spheres with a 6‐mm radius centered on the following coordinates: bilateral A1 (−51, −22, 8; 54, −10, 2; MNI) (Samson et al. 2011; Stickel et al. 2019), bilateral V1 (18, −94, 5; −1, −57, 20; MNI) (Shen et al. 2016; Stickel et al. 2019), bilateral S1 (−10, −4,8; 13, −22, 5; MNI), and bilateral O1 (−30, 18, 6; 38, 14, −4; MNI) (Figure 1a). To elaborate on the intra‐FCs of the primary sensory network, we calculated ROI‐wise connectivity (Fan et al. 2017; Nair et al. 2013; Yang et al. 2024). ROI‐wise connectivity was measured by extracting the average time courses for each ROI, calculating the correlation coefficients between each ROI pair, and applying the Fisher *z*‐transformation.

### 2.4 Multisite Effect Correction

To account for site, collection time, and data acquisition parameter variability across each of the data collections in ABIDE I, we employed ComBat (https://github.com/Jfortin1/ComBatHarmonization) in MATLAB version R2022a to correct the multisite effect for within‐network FC. It has been shown to perform well in harmonizing neuroimaging measurements (Johnson et al. 2007; Radua et al. 2020), particularly in its application to FC (Nielson et al. 2018).

### 2.5 Statistical Analysis

Statistical analysis was performed using SPSS version 26.0 (https://www.ibm.com/analytics/spss‐statistics‐software) and SPM12.

Demographic characteristics and clinical data were compared between the ASD and TD groups using SPSS version 26.0. Unpaired two‐sample *t*‐tests were employed to compare groups of continuous variables, such as age and FIQ score, whereas chi‐square tests were used for categorical variables, such as sex.

Analyses of ROI‐wise connectivity were conducted using SPSS version 26.0. All *z* values indicating ROI pair correlations were subjected to a two‐sample *t*‐test. The results were corrected for multiple comparisons using a false discovery rate (FDR) correction (corrected *p* < 0.05).

Abnormal ROI‐wise FC, extracted as the average *z*‐score of all voxels within each surviving cluster, was used in the subsequent correlation analysis. Social symptom severity was assessed using five diagnostic scores: the Autism Diagnostic Interview‐Revised (ADI‐R) total, social, verbal, and repetitive behavior scores and the Autism Diagnostic Observation Schedule (ADOS). Higher scores on these scales indicate greater severity of ASD‐related social impairment.

The effects of sex and age on aberrant FC were further analyzed using analysis of variance followed by Bonferroni's post hoc correction. Statistical significance was set at *p* < 0.05.

## 3 Results

### 3.1 Demographic Information

The demographic and clinical characteristics of the participants are summarized in Table 1. There were no group differences in terms of sex, age, or IQ.

**TABLE 1:** Demographic and clinical characteristics of ASD and TD groups.

Variable	Mean (SD)		Statistics
	ASD (*n* = 105)	TD (*n* = 132)	
Sex (male/female)	94/11	107/25	*χ* ^2^ = 3.251, *p *= 0.071
Age	13.96 (0.42)	14.7 (0.40)	*t *= −1.413, *p *= 0.159
FIQ	108.57 (1.52)	110.4 (1.12)	*t *= −0.967, *p *= 0.335
VIQ	106.58 (1.59)	110.16 (1.16)	*t *= −1.183, *p *= 0.071
PIQ	108.79 (1.53)	108.26 (1.23)	*t *= −0.271, *p *= 0.159
ADIR total score	41.16 (0.94)	∖	∖
ADIR social	19.66 (0.52)	∖	∖
ADIR verbal	15.67 (0.47)	∖	∖
ADIR RRB	5.83 (0.22)	∖	∖
ADOS score	11.13 (0.34)	∖	∖

Abbreviations: ADIR RRB, Autism Diagnostic Interview‐Revised Repetitive Behaviors; ADIR social, Autism Diagnostic Interview‐Revised social; ADIR verbal, Autism Diagnostic Interview‐Revised verbal; ADIR, Autism Diagnostic Interview‐Revised; ADOS, Autism Diagnostic Observation Schedule; ASD, autism spectrum disorder; FIQ, full‐scale intelligence quotient; SD, standard deviation; TD, typically developing.

### 3.2 ROI‐Wise FC Analysis

There were significant group differences in ROI‐wise FC between several pairs of regions (Figure 1b). Specifically, increased FC was demonstrated between the left A1 and right A1 (*p *= 0.005, Cohen's *d* = 0.66, 95% CI = [0.027, 0.135]), left O1 (*p *= 0.004, Cohen's *d* = 0.4, 95% CI = [0.022, 0.103]), left S1 (*p *= 0.008, Cohen's *d* = 0.35, 95% CI = [0.015, 0.099]), and right S1 (*p *= 0.002, Cohen's *d* = 0.4, 95% CI = [0.021, 0.108]) in ASD compared to TD. Increased FC was also observed between the right A1 and left O1 (*p *= 0.003, Cohen's *d* = 0.52, 95% CI = [0.02, 0.104]), left S1 (*p *= 0.011, Cohen's *d* = 0.3, 95% CI = [0.013, 0.093]), and right S1 (*p *= 0.004, Cohen's *d* = 0.56, 95% CI = [0.021, 0.108]) in ASD compared to TD. Increased FC between the right O1 and left O1 (*p *= 0.003, Cohen's *d* = 0.3, 95% CI = [0.006, 0.127]; Figure 1c) was observed in ASD compared to TD (abnormal FC patterns of each age stage, Figures S1–S3).

### 3.3 Brain–Behavior Associations

**FIGURE 2:**
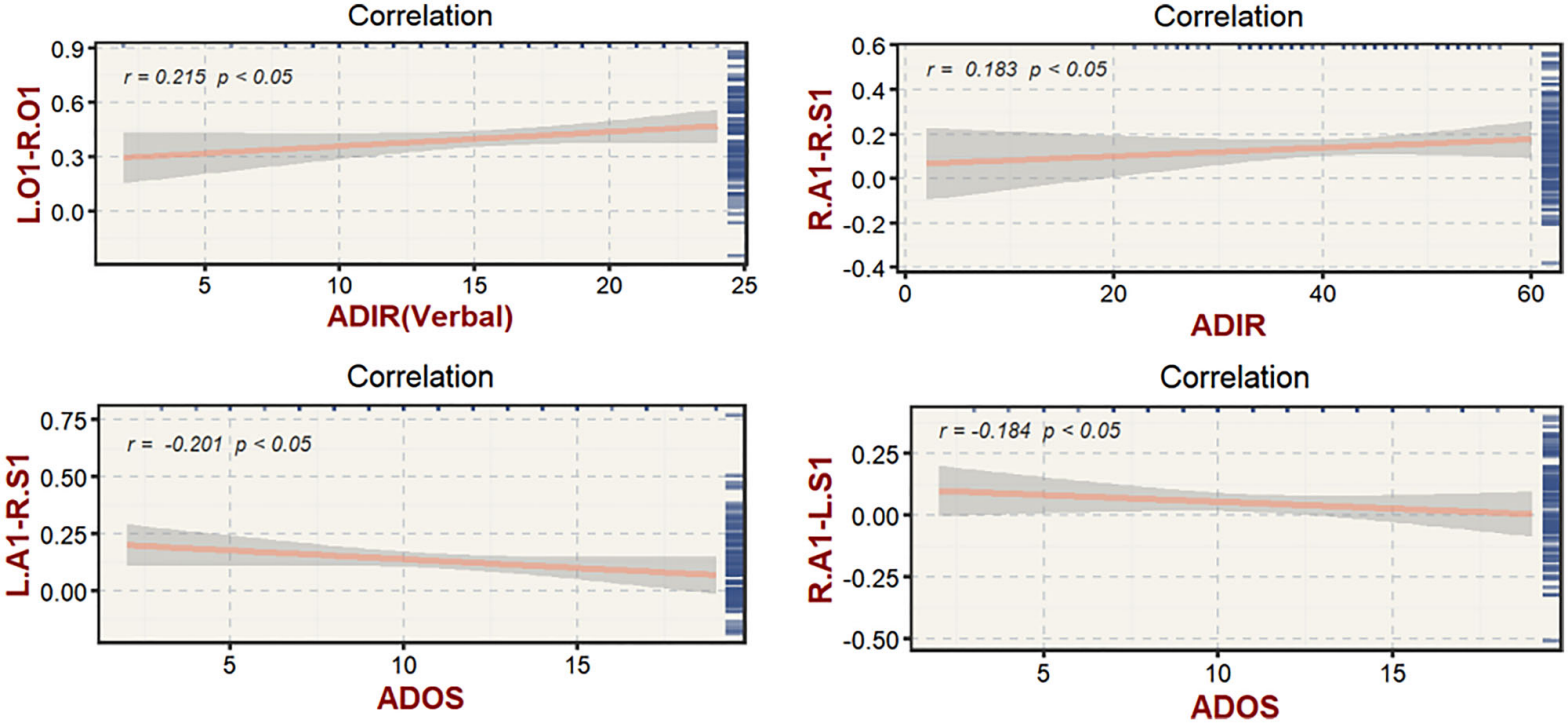
**Correlation analysis between aberrant ROI‐wise FCs and symptom severity**. Scatter plots showing the relationship between aberrant FCs and ADI‐R/ADOS scores. Dark gray shading indicates 95% confidence intervals. ADI‐R, Autism Diagnostic Interview‐Revised; ADOS, Autism Diagnostic Observation Schedule.

ADI‐R (verbal) scores were positively correlated with the FC of the left O1–right O1 (*r *= 0.215, *p *= 0.035), whereas ADI‐R (total) scores were positively correlated with the FC of the right A1–right S1 (*r *= 0.183, *p *= 0.037). ADOS scores were negatively correlated with the FC of the right A1–left S1 (*r *= −0.184, *p *= 0.036) and left A1–right S1 (*r *= −0.201, *p *= 0.025; Figure 2; more detail about correlation analyses on each subscale of the ADI‐R, Table S2).

### 3.4 Sex and Age Effects

**FIGURE 3:**
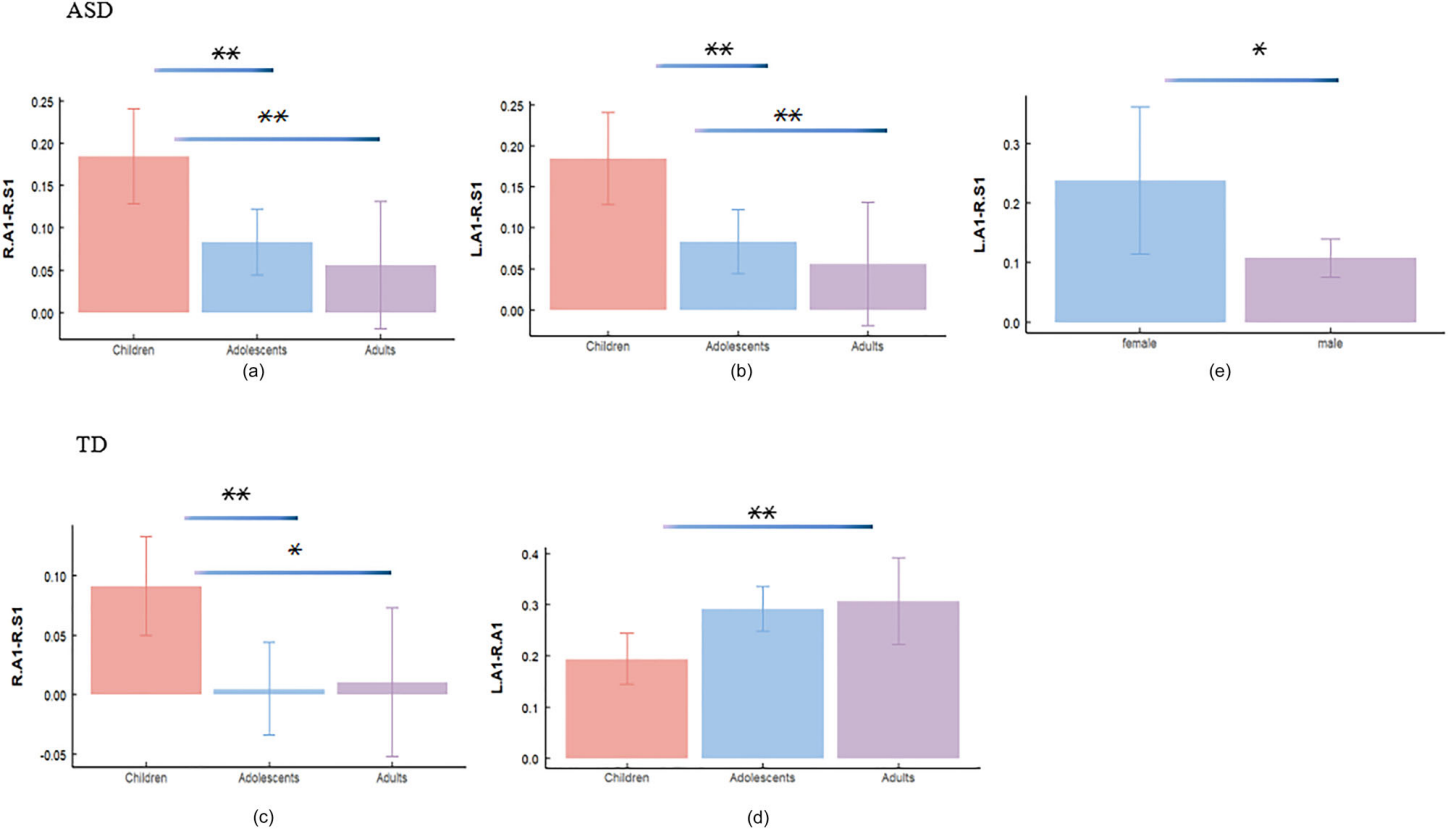
**Effect of age and sex on abnormal functional connectivity in ASD and TD**: (a and b) main effect of age on FC in ASD (from left to right: children, adolescents and adults in sequence); (c and d) main effect of age on FC in TD (from left to right: children, adolescents and adults in sequence); (e) main effect of sex on FC in ASD (from left to right: female, male) (*p < 0.05; **p < 0.01). ASD, autism spectrum disorder; TD, typically developing.

Age was a significant factor for FC. In ASD, the FC between the right A1 and right S1 exhibited a significant main effect of age (*F*(2, 0.158) = 6.381, *p *= 0.002, partial *η* ^2^ = 0.317; Figure 3a), with children showing higher values than adolescents (*p *= 0.001) and adults (*p *= 0.004). FC between the left A1 and right S1 exhibited a significant main effect of age (*F*(2, 0.226) = 7.056, *p *= 0.001, partial *η* ^2^ = 0.452; Figure 3b), with children showing higher values than adolescents (*p *= 0.003) and adults (*p *= 0.004). In TD, the FC between the right A1 and right S1 exhibited a significant main effect of age (*F*(2, 0.161) = 4.901, *p *= 0.009, partial *η* ^2^ = 0.322; Figure 3c), with children showing higher values than adolescents (*p *= 0.004) and adults (*p *= 0.022). The FC between the right A1 and left A1 exhibited a significant main effect of age (*F*(2, 0.106) = 4.761, *p *= 0.01, partial *η* ^2^ = 0.212; Figure 3d), with children showing lower values than adolescents (*p *= 0.008) and adults (*p *= 0.008). Within‐group differences in sex were observed in the ASD group. There were significant group differences in the FC between the left A1 and right S1 (*p *= 0.013, Cohen's *d* = −0.76, 95% CI = [−0.232, −0.028]; Figure 3e), with females showing higher values than males, whereas no sex differences were found in the TD group (correlation results between FC and ADOS/ADI‐R scores for male and female participants with ASD, Table S3). Moreover, we conducted further statistical tests and observed a significant age‐by‐sex interaction (*F*(2, 0.074) = 3.208, *p *= 0.045, partial *η* ^2^ = 0.061). Children showed higher values than adolescents in males (*p *= 0.023), whereas no significant differences were found in females (Figure S4).

### 3.5 Validation

**FIGURE 4:**
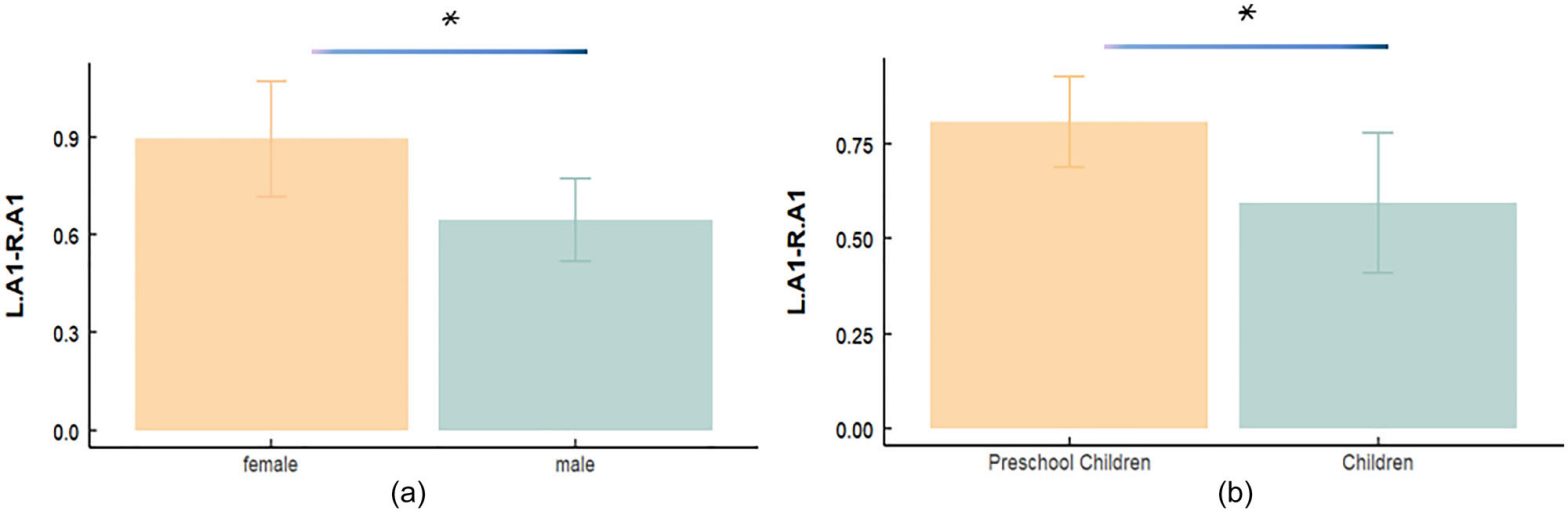
**Effect of sex and age on the abnormal functional connectivity in ASD**: (a) effect of sex on FC between L.A1 and R.A1 (from left to right: female, male); (b) effect of age on FC between L.A1 and R.A1 (from left to right: female, male).

A small autism dataset from two groups was analyzed. The first group comprised 30 individuals, 18 males and 12 females, with an average age of 3.3 years. The second group consisted of 23 individuals (20 males and 3 females) with an average age of 7.1 years. The results aligned with those of the large dataset (ABIDE). Age and sex were crucial factors influencing FC. Sex disparities were observed in the ASD group. A notable group difference in FC was detected between the left A1 and right A1 (*p *= 0.032, Cohen's *d* = −0.76, 95% CI = [−0.047, −0.023]; Figure 4a), with females showing higher values than males. However, no sex‐related differences were observed in the TD group. Regarding age, a significant group effect of FC was observed between preschool and school‐aged children in the left A1 and right A1 (*p *= 0.042, Cohen's *d* = −0.76, 95% CI = [0.008, 0.42]; Figure 4b).

## 4 Discussion

In this study, we initially performed a selection process on rs‐fMRI data from the ABIDE I dataset, obtaining rs‐fMRI data from 105 individuals with ASD and 132 TD individuals to explore abnormal FC patterns within the primary sensory networks of individuals with ASD and their correlation with clinical symptoms. Consistent with our initial hypothesis, individuals with ASD presented abnormal FC patterns in the primary network compared with TD individuals, which were associated with social and communication impairments. Furthermore, age significantly impacted the FC of the auditory and right somatosensory cortices. As age increases, the abnormal FC in autism shows an overall developmental trend that is fast at first and then slows. Sex also has an impact on the FC of the auditory and somatosensory cortices, as well as the FC of the olfactory and visual cortices. The FC of females was higher than that of males, and the results from the two‐site dataset collected in the laboratory were consistent with those from the multisite data, indicating the stability of our findings. These findings have the potential to identify new biomarkers for ASD.

Our exploration of FC within the primary sensory cortex revealed that individuals with ASD have heightened connectivity in contrast to the TD group, particularly with marked alterations in the FC between the auditory and somatosensory regions. Incorporating this finding with the “Intense World Theory” put forward by Markram and Markram (2010), we deduce that the enhanced intrinsic network connectivity observed in ASD might originate from over‐functionality within local neural microcircuits, encompassing hyperreactivity and hyper‐plasticity. Such over‐functionality can result in an exaggerated response and processing of sensory inputs within the primary sensory cortex, especially in the auditory and somatosensory domains, in individuals with ASD. In these individuals, the auditory cortex may be involved in the processing of sound‐related information, including speech and nonverbal vocalizations during social interactions (Cook et al. 2022). Abnormalities in the somatosensory cortex could potentially influence perception and response to tactile stimuli in individuals with ASD (Pan et al. 2022). Research has indicated that the somatosensory cortex of individuals with ASD displays aberrant neural network connectivity patterns at rest, which may be associated with social and communicative impairments (Iannone and García 2021). Additionally, significant alterations in FC within certain visual networks have been reported in children with ASD (Xu et al. 2019), and thalamocortical and insular bundles that convey somatosensory information have also been found to exhibit reduced structural integrity in autism (Failla et al. 2017). However, these studies mainly focused on higher cognitive processes and did not explore the implications of these FC changes within the primary sensory cortex.

Correlational analysis using clinical scales suggests that the pattern of abnormal FC found in individuals with ASD may be useful for predicting clinical severity. Our study affirmed that individuals with higher ADI‐R (verbal) scores may demonstrate higher FC, whereas those with higher ADOS scores may show lower FC. The relationship between clinical scales, such as the ADOS and ADI‐R, and FC has been verified in some studies (Lebersfeld et al. 2021; Tong et al. 2024). However, the strength of these correlations was moderate. We speculate that this may be attributed to the fact that, compared to primary sensory networks, the connectivity of higher order cognitive networks more directly reflects the behavioral symptoms of children with autism, such as social functioning and social cognition. Yoon et al. (2022) found that abnormal FC of the DMN was significantly correlated with symptom scales in children with autism. Meanwhile, neuroimaging studies have demonstrated that anatomically defined subgroups can enhance the predictive power of ASD symptom severity, suggesting that individual differences and the interplay between genetic and environmental factors, including inflammation, immune responses, oxidative stress, and mitochondrial dysfunction, may affect the relationship between ASD clinical symptoms and abnormal FC (Buch et al. 2023; Zhuang et al. 2024). Sex may also be a confounding factor affecting the correlation between ASD FC and symptom scales. This may be related to the Female Protective Effect in ASD (Werling and Geschwind 2013). Cola et al. (2022) found that girls used more “socially focused” words, specifically regarding friends, than boys despite the samples being matched for intellectual functioning and autism symptom severity. This may result in the inability of females to truly demonstrate their abilities during symptom assessment.

Our research found that children with ASD show overconnectivity in brain networks during the school‐age period, whereas underconnectivity may commence in adolescence and persist into adulthood. However, in TD individuals, the cellular processes of synaptic growth, pruning, and myelination occur throughout childhood and into adulthood, with corresponding changes in brain anatomy, function, and connectivity, particularly during puberty (Stiles and Jernigan 2010). Generally, children reach their peak during early adolescence and then decline (Kaczkurkin et al. 2019). Studies have shown that FC abnormalities in individuals with ASD may undergo specific changes with age, which may be associated with neurodevelopmental processes in individuals with ASD (Haghighat et al. 2021; Padmanabhan et al. 2013), highlighting the importance of early interventions. This result supports the EBO in children with ASD (Raznahan et al. 2013) that suggests children with ASD exhibit excessive brain growth in early life stages.

Our findings are consistent with several previously published FC studies focusing on sex differences in ASD, which have generally reported that females with ASD show increased connectivity in numerous brain regions compared to males with ASD (Smith et al. 2019), possibly attributed to hormones (Bos et al. 2016). Alexandru hypothesized that any abnormality in sex hormones predisposes an individual to specific molecular malfunctions that alter brain connectivity (Niculae and Pavăl 2016). The Extreme Male Brain Theory suggests that the autistic brain is an extreme variant of the male brain (Baron‐Cohen 2002) due to abnormally high exposure to testosterone between Weeks 8 and 24 of gestation (Knickmeyer and Baron‐Cohen 2006). Moreover, Sie et al. showed that sex hormones, particularly estrogen, exert a significant impact on brain FC during early adulthood in females. ASD has long been considered a predominantly male disorder, which may affect identification and diagnosis in women. Females with ASD may have differences in ASD symptom expression compared to men due to social and biological differences and therefore do not follow the prototypical male profile of ASD symptoms.

We further observed interactions between the differences in FC according to sex and age. Studies investigating age‐related brain differences across a broad age range have suggested distinct neurodevelopmental patterns in females and males with ASD. Research shows that sex differences in the DMN vary with age; for example, intrinsic connectivity in the DMN declines faster in males than in females as they age (Scheinost et al. 2015). Wagner et al. (2019) used a longitudinal co‐twin design and found that females with ASD showed a peak in maternally reported ASD symptom severity during early adolescence and declined thereafter; however, males with ASD and unaffected male and female siblings showed modest linear increases in autistic traits from childhood to early adulthood. From the perspective of cross‐disease research, we have observed certain commonalities in the age and sex effects on brain FC between individuals with attention deficit/hyperactivity disorder (ADHD) and those with ASD (Nikolaidis et al. 2022; Wu et al. 2023). Similar to individuals with ASD, those with ADHD exhibit abnormal FC patterns as they grow older. Hong et al. (2024) reported an age‐associated increase in the number of regions with enhanced FC within the DMN and attention network in an ADHD group. Furthermore, female individuals with ADHD are also affected by PET in terms of symptom manifestation. Typically, females are only diagnosed when their symptoms are extremely severe (Siddiqui et al. 2024). Research from a cross‐disease perspective not only reflects the potential commonalities among different mental disorders at the neurobiological level but also offers the possibility of precisely identifying universal biomarkers.

### 4.1 Limitations

This study had several limitations. First, the impact of confounding factors on the correlation between FC and clinical scores was not excluded. Although this correlation between clinical scale scores and abnormal FC was moderate, the statistical significance proved the existence of a connection. Future studies should exclude the effect of confounding factors on the correlation between symptom scores and FC. Second, the relatively small sample size and poor matching by age and sex in the validation dataset may limit generalizability. Consequently, caution must be exercised when extrapolating the conclusions to a wider population. Future research endeavors that incorporate a larger and more sex‐balanced validation dataset will further enhance the robustness and universality of the research findings, providing a more solid and reliable foundation for related fields. Third, although the impacts of sex and age were analyzed in the present study, the insufficient number of female participants restricts the interpretation of the results. Future studies should incorporate more data from females. Finally, although differences in FC appear promising, the usefulness of these measures for individual diagnosis or treatment planning remains uncertain. Further validation and large‐scale studies are required to confirm the robustness and generalizability of these biomarkers.

## 5 Conclusion

Research suggests that abnormal FC exists in the primary sensory cortex of individuals with ASD. These connections are associated with clinical outcomes and may lead to impairments in advanced cognitive function. In addition, the primary sensory cortex of individuals with ASD experiences excessive growth during the early stages of the disease and demonstrates unbalanced sexual development. These findings may help identify potential biomarkers of ASD. Our results were validated using dual‐site datasets collected in the laboratory and indicated a certain level of stability. However, it must be acknowledged that, at the present stage, the effectiveness of these indicators for individual diagnosis or treatment planning remains speculative. Further verification and larger scale studies are required to establish the robustness and generalizability of these biomarkers. Nevertheless, the findings of this study can fill the existing gaps in research on the primary sensory cortex in the field of autism and offer some assistance for future intervention and diagnosis of ASD.

## Author Contributions

**Yanan Zhang**: investigation, formal analysis, writing–review and editing, methodology, conceptualization, validation, writing–original draft, software, visualization. **Quan Zhou**: data curation, writing–original draft, writing–review and editing, investigation. **Limei Gao**: data curation, supervision. **Jingwen Li**: data curation, investigation. **Hong Li**: data curation; supervision, resources. **Gong‐Jun Ji**: software, methodology, supervision. **Hua Yang**: investigation. **Enze Wang**: investigation. **Kai Wang**: project administration, resources. **Dandan Li**: resources, project administration, funding acquisition.

## Ethics Statement

The study protocol was approved by the Institutional Review Board of Anhui Medical University.

## Consent

The participants and their parents were asked to sign an informed consent prior to their participation in the study.

## Conflicts of Interest

The authors declare no conflicts of interest.

### Peer Review

The peer review history for this article is available at https://publons.com/publon/10.1002/brb3.70363

## Supporting information



Supporting Information

## Data Availability

The data that support the findings of this study are available on request from the corresponding author upon reasonable request.
