# Supplementary material for: Abnormal Functional Connectivity of the Primary Sensory Network in Autism Spectrum Disorder: Sex Differences, Early Overdevelopment, and Clinical Significance
Source: Brain Behav. 2025 Mar 23;15(3):e70363. doi: 10.1002/brb3.70363 (PMC11930894; doi:10.1002/brb3.70363)
Supplement: Supplementary file 1 — Supporting Information [file BRB3-15-e70363-s001.docx]

**Supplementary materials**

Supplementary materials about our manuscript entitled **“*Abnormal functional connectivity of the primary sensory network in autism spectrum disorder: sex differences, early overdevelopment, and clinical significance*”**

**Methods**

**MRI data acquisition**

ABIDE:

Based on the site data obtained from the ABIDE official website, we have meticulously collated and extracted the basic imaging acquisition sequences from the eight sites where all the subjects were sourced. However, due to the lack of uniformity in information and the incomplete data across different sites, we were unable to present the acquisition sequences of certain sites. We understand the importance of this information for ensuring methodological transparency and reproducibility, and we apologize for this limitation. The information table is as follows:

**Table S1.** **ABIDE---MRI data acquisition**

| Site | Functional pulse sequence | Structural pulse sequence |
| --- | --- | --- |
| STANFORD | REST spiral in-out | Unknown |
| SDSU | GRE-EPI | SPGR |
| TRINITY | FFE-EPI | FFE |
| NYU | EPI | Unknown |
| LEUVEN-1 | FFE-EPI | FFE |
| PITT | Unknown | MPRAGE |
| UCLA1 | Unknown | ADNI_MPRAGE |
| UCLA2 | Unknown | ADNI_MPRAGE |

**Validation dataset from our lab's----MRI data acquisition**

**MRI data acquisition-Site 1**

All functional and structural MRI datasets were collected at the Anhui Hospital Affiliated to the Pediatric Hospital of Fudan University using a 3.0-T scanner (Philips Ingenia CX). Functional images (240 volumes) were obtained using an echo-planar imaging sequence (repetition/echo time: 2000/30 ms; flip angle: 90°). Images of 46 transverse slices (field of view: 192 mm × 192 mm; matrix: 64 × 64; slice thickness: 3 mm with no inter-slice gap; voxel size: 3 mm × 3 mm × 3 mm) were acquired parallel to the anteroposterior commissure line. Subsequently, high spatial resolution T1-weighted anatomic images were acquired with the following parameters: repetition/echo time: 6.77/3.07 ms; flip angle: 8°; field of view: 256 × 256 mm^2^; matrix: 256 × 256; slice thickness: 1 mm with no inter-slice gap; voxel size: 1 × 1 × 1 mm^3^; 176 slices.

**MRI data acquisition-Site 2**

All functional and structural MRI datasets were collected at the University of Science and Technology of China (Hefei, China) using a 3.0-T scanner (Discovery 750; GE Healthcare, Milwaukee, WI, USA). Functional images (217 volumes) were acquired using an echo planar imaging sequence (repetition/echo time, 2400/30 ms; flip angle, 90º). Images of 46 transverse sections (field of view: 192×192 mm^2^; matrix: 64 × 64; section thickness: 3 mm without intersection gap; voxel size: 3 mm×3 mm×3 mm) were acquired parallel to the anteroposterior commissure line. Sagittally oriented high-resolution T1-weighted anatomical images were obtained using magnetization-prepared rapid gradient-echo sequence (repetition/echo time, 8.16/3.18 ms; flip angle, 12º; field of view, 256×256 mm^2^; 256×256 matrix; section thickness, 1 mm, without intersection gap; voxel size, 1×1×1 mm^3^; 188 sections).

**Results:**

**ROI-wise functional connectivity analysis**(Abnormal FC patterns of each age stage)


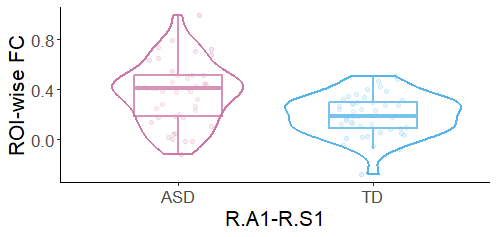


* *p=0.019*


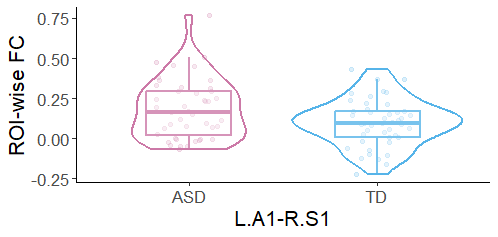


* *p=0.019*


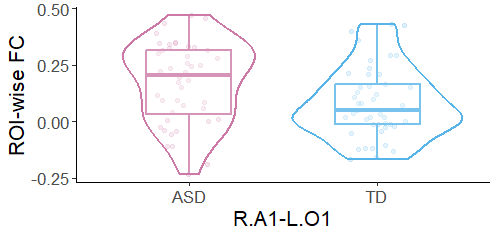


** *p=0.001*


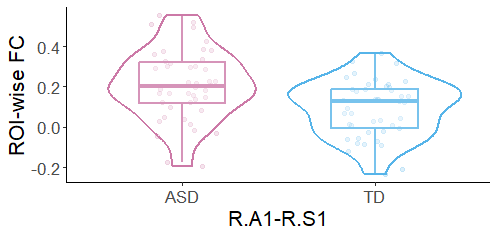


** *p=0.001*


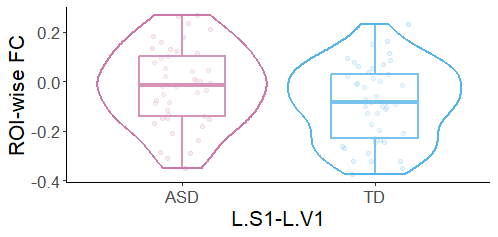

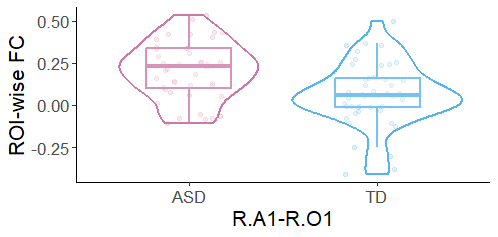


** *p=0.001*

* *p=0.044*

**Figure S1.** **Aberrant ROI-wise FC in childhood.**


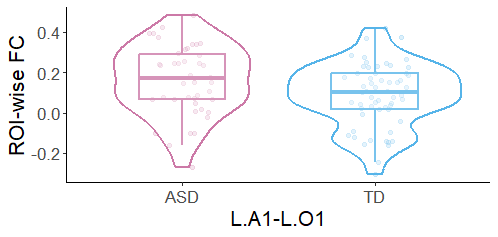

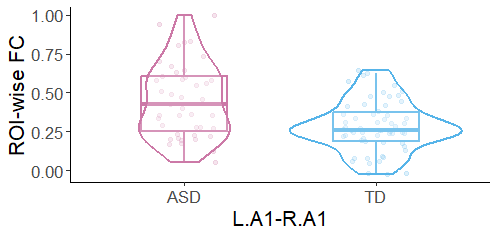


** *p=0.001*

* *p=0.022*


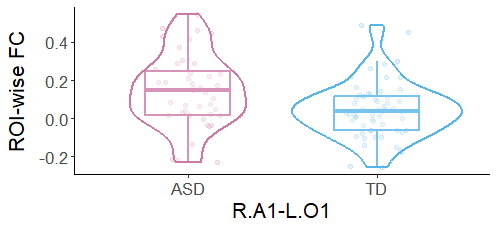


** *p=0.0087*


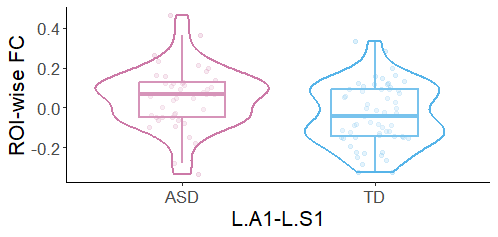


** *p=0.008*


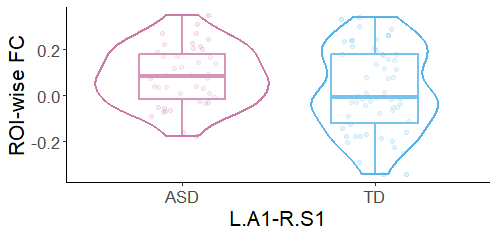


** *p=0.008*


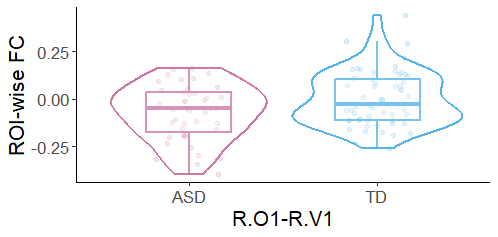


* *p=0.015*


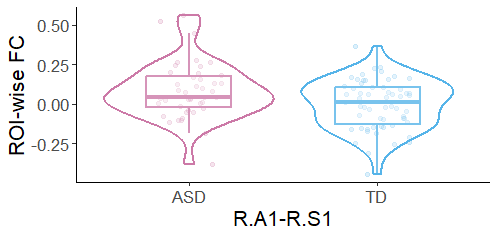


* *p=0.014*

**Figure S2. Aberrant ROI-wise FC in adolescence.**

* *p=0.03*


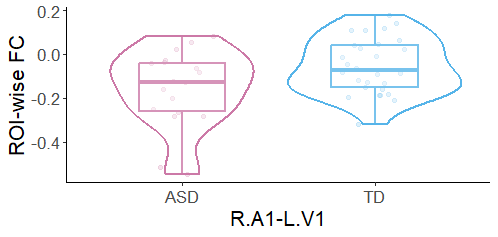


* *p=0.03*


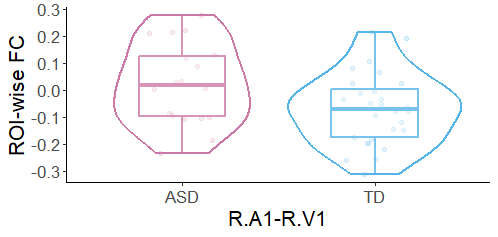


**Figure S3. Aberrant ROI-wise FC in adulthood.** Significant ROI-wise FCs. Box plots indicate the median and quartiles with whiskers indicating minimum and maximum values (pink represent ASD; blue represent TD). The violin plot outlines illustrate kernel probability density.(FDR correction. **p* < 0.05; ***p* < 0.01). L.A1,the left primary auditory cortex; R.A1,the right primary auditory cortex; L.V1, the left primary visual cortex; R.V1, the left primary visual cortex; L.S1, the left primary somatosensory cortex; R.S1, the primary right somatosensory cortex; L.O1, the left primary olfactory cortex; R.O1, the right primary olfactory cortex

**Brain-behavior associations**

**Table S2. Correlation analyses on each subscale of the ADI-R**

|  | **ADI-R Total** | | **ADI-R Social** | | **ADI-R Verbal** | | **ADI-R RRB** | | **ADOS Total** | |
| --- | --- | --- | --- | --- | --- | --- | --- | --- | --- | --- |
|  | *r* | *p* | *r* | *p* | *r* | *p* | *r* | *p* | *r* | *p* |
| **L.O1-R.O1** | 0.139 | 0.177 | 0.035 | 0.732 | 0.215* | 0.035 | 0.038 | 0.711 | -0.134 | 0.097 |
| **R.A1-R.S1** | 0.183* | 0.037 | 0.090 | 0.191 | 0.165 | 0.054 | 0.070 | 0.249 | -0.05 | 0.315 |
| **L.A1-R.S1** | 0.076 | 0.232 | 0.027 | 0.396 | 0.057 | 0.290 | 0.063 | 0.272 | -0.201* | 0.036 |
| **R.A1-L.S1** | -0.002 | 0.492 | 0.037 | 0.360 | -0.04 | 0.350 | -0.069 | 0.253 | -0.184* | 0.025 |

**Gender and age effects**

|  | **ADI-R Total** | | **ADI-R Social** | | **ADI-R Verbal** | | **ADI-R RRB** | | **ADOS Total** | |
| --- | --- | --- | --- | --- | --- | --- | --- | --- | --- | --- |
| **Males** | ***r*** | ***p*** | ***r*** | ***p*** | ***r*** | ***p*** | ***r*** | ***p*** | ***r*** | ***p*** |
| **L.A1-R.S1** | 0.018 | 0.434 | -0.001 | 0.497 | 0.022 | 0.420 | 0.011 | 0.461 | -0.208* | 0.027 |
| **Females** | ***r*** | ***p*** | ***r*** | ***p*** | ***r*** | ***p*** | ***r*** | ***p*** | ***r*** | ***p*** |
| **L.A1-R.S1** | -0.122 | 0.369 | -0.340 | 0.169 | -0.159 | 0.330 | 0.263 | 0.231 | 0.354 | 0.158 |

**Table S3. Correlation analyses between the FC of male and female participants with ASD**

In male patients with ASD, our findings revealed a negative correlation between the ADOS scores and the FC of the left A1-right S1 (r=-0.208, *p*=0.027; see **Table S3**). However, no significant correlations were observed with other scales. In contrast, for female patients, no significant correlations were detected across all scales.

Moreover, we conducted further statistical tests and observed a significant age-by-sex interaction(F (2, 0.074)=3.208, *p*=0.045, partial η^2^=0.061). Children showed higher values than adolescents in males (*p*=0.023), whereas no significant differences were found in females(**Figure S4**).


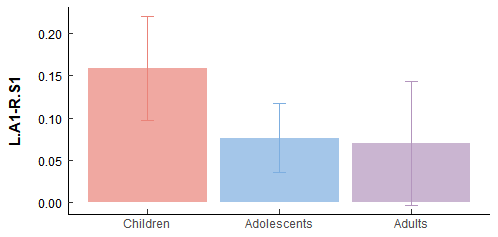

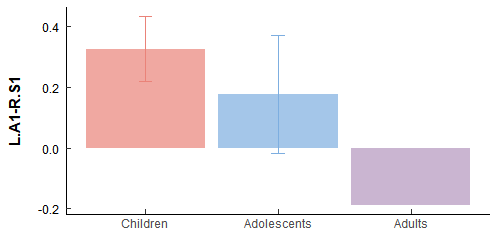


male

female

* *p=0.023*

**Figure S4.** **Main effect of age and age-by-gender interaction in FC between the left A1 and right S1.**
